# Effective optimization of irrigation networks with pressure-driven outflows at randomly selected installation nodes

**DOI:** 10.1038/s41598-023-45844-3

**Published:** 2023-11-06

**Authors:** E. Creaco, G. Barbero, A. Montanaro, M. Reduzzi

**Affiliations:** 1 Dipartimento di Ingegneria Civile e Architettura, Università degli Studi di Pavia, Pavia, Italy; 2 Consorzio di Bonifica della Media Pianura Bergamasca, Bergamo, Italy

**Keywords:** Environmental sciences, Hydrology, Engineering

## Abstract

This paper presents an innovative methodology for the design of pressurized irrigation networks. Compared to other methodologies proposed in the scientific literature, it features three novel aspects: (i) construction of peak demand scenarios based on the random selection of installation nodes for hydrant heads available in each sector of irrigated properties; (ii) realistic hydraulic modelling of outflows from hydrant heads by means of the pressure driven approach; and (iii) adoption of linear constraints to enforce the telescopic property in the distribution of diameters from the source towards the external areas of the network in the optimized design. The applications of the methodology to the real network serving an irrigated area of 750 ha in Northern Italy proved that the aspects (i) and (ii) contribute to the accurate modelling of the current network while highlighting its hydraulic deficiencies. The adoption of the linear constraints described in (iii) in the context of the bi-objective genetic optimization of network diameters resulted in the speeding up of the algorithm convergence. The results show how decision makers can choose the ultimate configuration based on budget considerations from the trade-off solutions obtained between installation costs and hydraulic performance, considering network layouts with different level of topological redundancy.

## Introduction

Problems of water scarcity plague large areas in the world, especially arid and semiarid areas such as the Mediterranean region. These problems cause serious consequences in countries like Spain, Italy and Portugal, which are rendered the most water-consuming countries in the European Union by the presence of a strong agricultural sector. In fact, 70% of the world’s freshwater withdrawals are nowadays earmarked for agriculture and this percentage is expected to increase, in order to meet the growing population’s demand for food and energy (biofuels)^1^. Over the last decades, among the various practices implemented by irrigation managers to mitigate the effects of water scarcity, open channel irrigation systems with high leakage rates have been replaced with pressurized water networks^2^, which operate either on demand or based on rotation delivery scheduling. Therefore, methodologies have recently been proposed to design/rehabilitate pressurized irrigation networks, that is to obtain highly effective networks able to meet desired water demands and service pressure with limited installation cost.

Among the various methodologies proposed in the scientific literature, Reca and Martinez^3^ proposed a single objective genetic algorithm to design pressurized irrigation networks. Farmani et al.^4^ made use of a modified bi-objective genetic algorithm, in which the algorithm operators were modified to improve the effectiveness. The Authors proved that their algorithm yields better numerical performance than the linear programming in the design of branched irrigation systems operating on-demand or based on rotation delivery scheduling. Fernández García et al.^5^ compared two bi-objective algorithms, based on genetic algorithms and linear programming respectively, in the rehabilitation of pressurized irrigation networks with the aim to increase energy efficiency. Finally, Rubio-Castro et al.^6^ proposed a mathematical programming model for the optimal design of integrated agricultural water networks, based on a superstructure that includes all configurations in terms of use, reuse and regeneration of water over the territory.

Other works addressed layout optimization along with pipe design. In this context, Lamaddalena et al.^7^ proposed a sequential algorithm for identifying the loops to be closed to improve the hydraulic performance, whereas Fouial et al.^8^ implemented loop re-closure in the framework of the multi-objective genetic optimization. Furthermore, Masoumi et al.^9^ presented a multi-objective methodology based on the Max–Min ant optimization to design both layout and pipe diameters in pressurized irrigation networks operating on-demand.

Though being outstanding contributions to the field, the scientific works mentioned above and focussed on the optimization of pressurized irrigation networks are all based on the simplifying assumption of demand-driven outflows from hydrant heads used for irrigation. In other words, nodal demands are assigned as constant values independent of service pressure. The literature of urban water distribution systems has shown that the pressure driven approach, which models nodal outflows as a growing function of service pressure, yields a more realistic representation of network behaviour^10–14^, though causing trouble in the convergence of hydraulic modelling algorithms in some cases^15^. The use of pressure-driven modelling is quite limited in the context of irrigation networks (e.g., see^16,17^) with no focus on the optimization context. This gap is bridged in the present paper, in which a methodology is proposed for the pressure-driven modelling of hydrant outflows for use in the optimization/design context.

The methodology features another novel aspect related to the reconstruction of peak demand scenarios. In the context of modelling demand scenarios^18,19^, methodologies were proposed in the scientific literature (e.g., see^17,20,21^) for demand estimation in pressurized irrigation networks operating on demand. These methodologies implement irrigation management practices followed by farmers and computational procedures and enable the soil water balance and the irrigation events for all cropped fields supplied by each delivery hydrant in a distribution network to be considered. In the present work, peak demand scenarios are reconstructed based on representative combinations of potentially simultaneous hydrant openings in pressurized irrigation networks subdivided into sectors of properties, inside each of which farmers use the available hydrant heads based on rotation delivery scheduling. Soil water balance is ignored as each peak demand scenario is meant to reproduce a plausible instantaneous demand forcing condition to test the hydraulic performance of the network in the optimization context, not a temporal sequence of demands at network nodes.

A third final novel aspect concerns the numerical efficiency of the optimization and is based on the enforcement of the telescopic property of pipe diameters for speeding up the convergence of genetic algorithms in network design.

The following sections report case study, methodology, results of the application and concluding remarks.

## Case study

**Figure 1:**
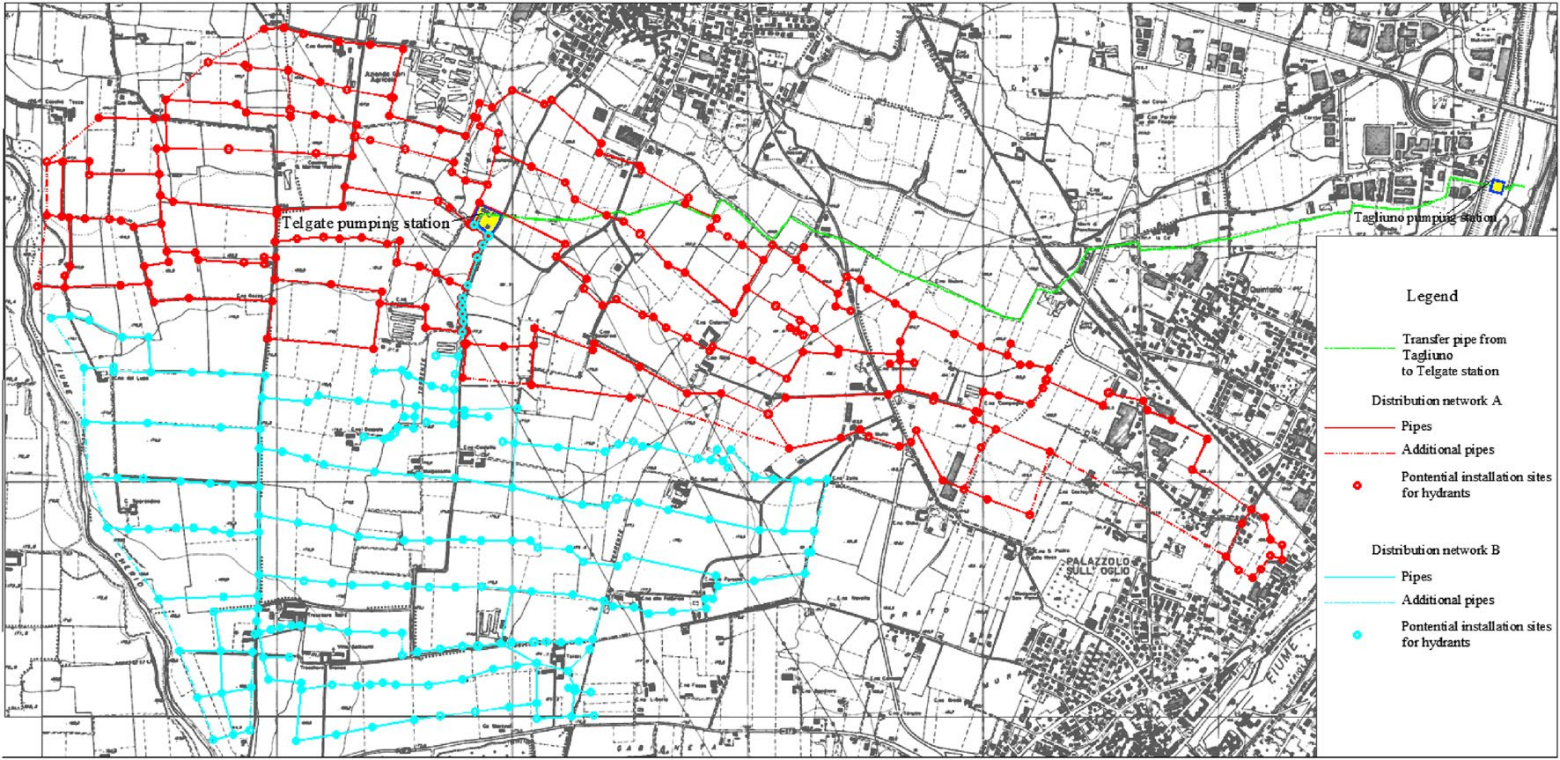
Plan view of the water transfer and distribution network in the case study area.

The water transfer and distribution network of Telgate is managed by the Consorzio di Bonifica della Media Pianura Bergamasca, hereinafter called “Consortium”, and serves with pressurized flow an area of about 750 ha, inside the towns of Telgate, Bolgare, Palosco, Palazzolo S/O, Grumello del Monte, Castelli Calepio, in the province of Bergamo. As is shown in Fig. 1, the network featuring a total length of 54.3 km is made up of two parts: the northern or high-altitude network (Network A) and the southern or low-altitude network (Network B) with a total length of 30.1 km and 24.2 km, respectively. The two networks are disconnected from each other and are both fed by the Telgate pumping station, in which eight pumps are present, three ordinary and one back-up pumps for either network. The upstream tank of the station has a head of 180 m above sea level and receives through a transfer pipe water flows withdrawn from the river Oglio, in correspondence to an intake located in Tagliuno (Castelli Calepio), while respecting the water concession water discharge of 600 L/s, which is reduced to 400 L /s under conditions of drought.

**Figure 2:**
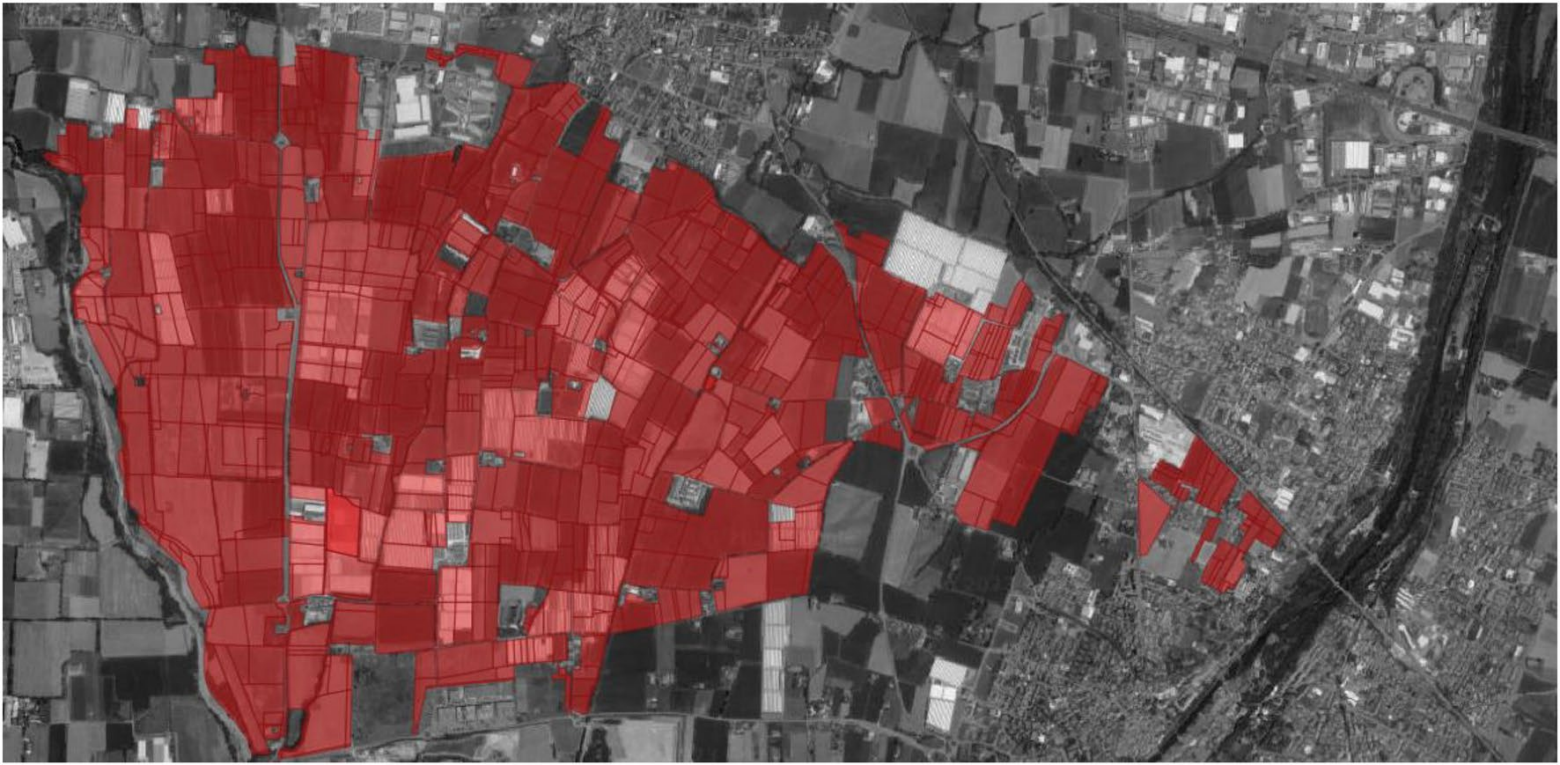
Properties of the Consortium highlighted with polygons filled with red colour. Map created with QGIS 3.28.3-Firenze and ESRI Satellite QuickMapServices.

In the served area, there are 924 irrigated properties (Fig. 2) grouped into 63 sectors. Each sector is assigned by the Consortium an irrigation water discharge *Q*_*irr*_ equal to the product of its area in ha and the Consortium specific discharge *d*_*irr*_ = 0.5 L/(s × ha), and a number of hydrant heads *N*_*hyd*_ = floor(max(*Q*_*irr*_/5, 1)), in which “floor” indicates the rounding to the lower integer.

**Figure 3:**
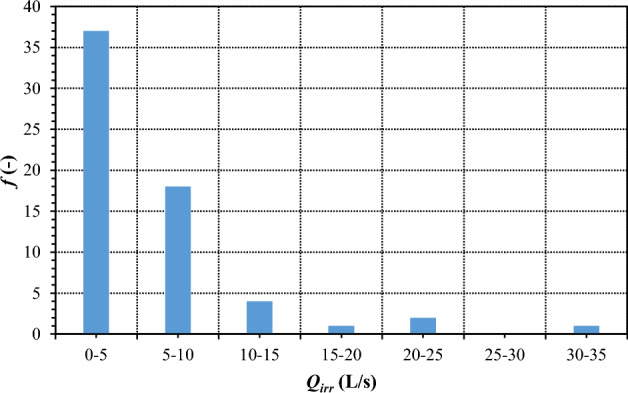
Frequency *f* of consortia for each class of *Q*_*irr*_.

The graph in Fig. 3 presents the frequency analysis of the values *Q*_*irr*_ in Consortium sectors. Most sectors feature values of *Q*_*irr*_ lying between 0 and 5 L/s (*f* = 37) or between 5 and 10 L/s (*f* = 18), therefore receiving a single hydrant head from the Consortium. There are *f* = 4 sectors within the range between *Q*_*irr*_ = 10 and *Q*_*irr*_ = 15 L/s receiving two hydrant heads, *f* = 1 sectors within the range between *Q*_*irr*_ = 15 and *Q*_*irr*_ = 20 L/s receiving three hydrant heads, *f* = 2 sectors within the range between *Q*_*irr*_ = 20 and *Q*_*irr*_ = 25 L/s receiving four hydrant heads and finally *f* = 1 sectors within the range between *Q*_*irr*_ = 30 and *Q*_*irr*_ = 35 L/s receiving six hydrant heads. No sector exists with *Q*_*irr*_ between 25 and *Q*_*irr*_ = 30 L/s. Summing up, 80 hydrant heads are distributed in the served area. Inside each sector of properties, farmers can use the available hydrant heads to irrigate their properties based on rotation delivery scheduling.

The Consortium network is plagued by various operational and managerial problems, related to its poor performance and old age. In fact, it is not capable of guaranteeing the desired service pressure and includes numerous old asbestos cement elements to be urgently replaced, as is prescribed by the law. These problems spurred the Consortium to evaluate the redesign of the infrastructure, which is the subject of the present work carried out in three phases:

- Phase 0: Hydraulic analysis of the current network.
- Phase 1: Redesign of the network considering the same layout as the current network, with a total length of 54.3 km.
- Phase 2: Redesign of the network considering a modified 61.8 km long layout, obtained from the current layout by adding 30 new sites, indicated with a dotted line in Fig. 1, to close the external loops.

## Methodology

The methodology adopted in this work is made up of three elements described in detail in the following subsections, namely a procedure for the reconstruction of peak demand scenarios (3.1), the hydraulic modelling of the network (3.2) and a procedure for the optimization of pipe diameters (3.3). The first two methodological elements were used in all phases of work (phases 0, 1 and 2). The third element was used, instead, only in phases 1 and 2, aimed at network design.

### Construction of peak demand scenarios

**Figure 4:**
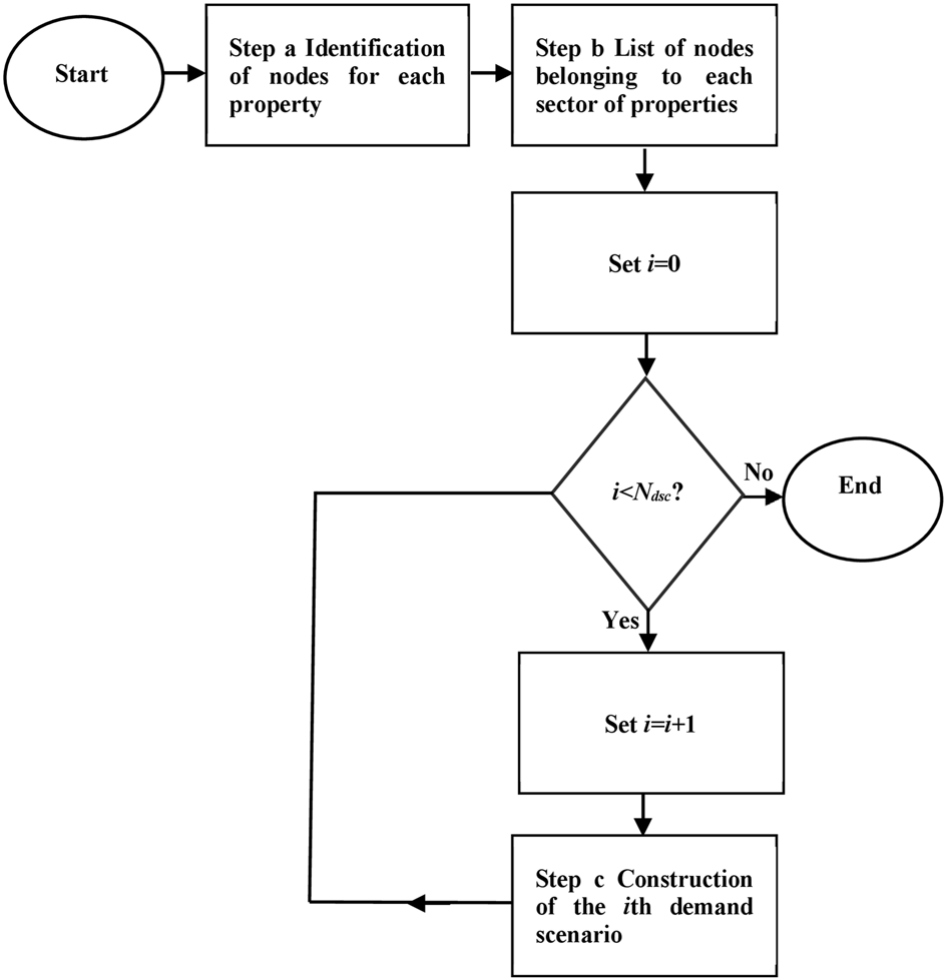
Flowchart for the construction of peak demand scenarios.

A novel algorithm was developed to construct a sample of *N*_*dsc*_ plausible peak demand scenarios, where a demand scenario is defined as an operational scenario in which all the 80 hydrant heads provided by the Consortium are simultaneously active. This algorithm is made up of three steps (flowchart in Fig. 4).

As Step a of the algorithm, the network nodes associated with each Consortium property were manually identified as potential hydrant locations by leaning upon the maps in Figs. 1 and 2. In this context, the generic node was associated with one of the Consortium properties if one of the following conditions held:

- The node was inside a property;
- If the node did not lie inside any properties, the node was associated with the spatially closest property.

The subsequent steps were carried out by using the programming language Matlab® 2023a^22^:

Step b—the network nodes associated with each sector of properties were first determined, by grouping the nodes associated with all the properties inside the sector;

Step c—A single demand scenario was then obtained by randomly selecting, for each hydrant head available in each sector of properties, the installation site among the list of nodes associated with that sector of properties.

Step c was reiterated as many times as the number *N*_*dsc*_ of demand scenarios to be generated.

Since the outflow at the generic node is assumed to lump the outflows occurring in the half pipes connected to this node from the hydraulic modelling viewpoint, the possibility of installing more than one hydrant head was considered in the algorithm at each network node.

### Hydraulic modelling of the network

The hydraulic model of the network was built inside the software EPANET 2.2^13^, which can solve the system of nodal mass conservation and pipe energy balance equations for each peak demand scenario generated as was shown in section “Construction of peak demand scenarios”. This new version of EPANET can model the pressure-driven behaviour of nodes in pressurized irrigation networks, without the implementation of artificial network elements (approach used by^17^).

For the pipes of the existing network, a Hazen Williams roughness coefficient of 100 was considered in the phase 0 of work, consistently with aged asbestos cement elements. For the pipes of the redesigned network, a Hazen Williams roughness coefficient of 150 was considered, consistently with plastic material, which is not destined to change with infrastructure growing old.

**Figure 5:**
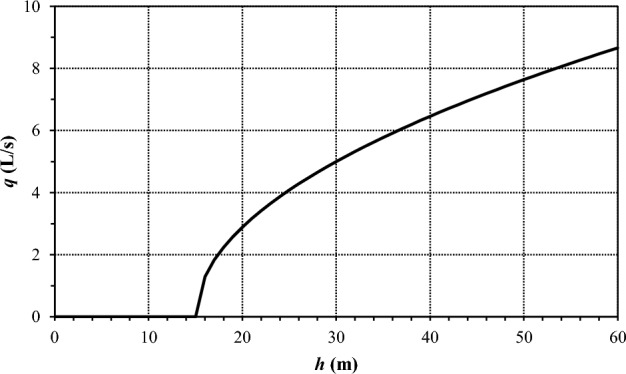
Relationship between outflow *q* and service pressure *h* at the generic hydrant head.

In correspondence to the generic hydrant head, the relationship between outflow and service pressure shown in Fig. 5 was considered, based on information gathered on the kind of devices provided by the Consortium and on how they are operated by the farmers.

**Figure 6:**
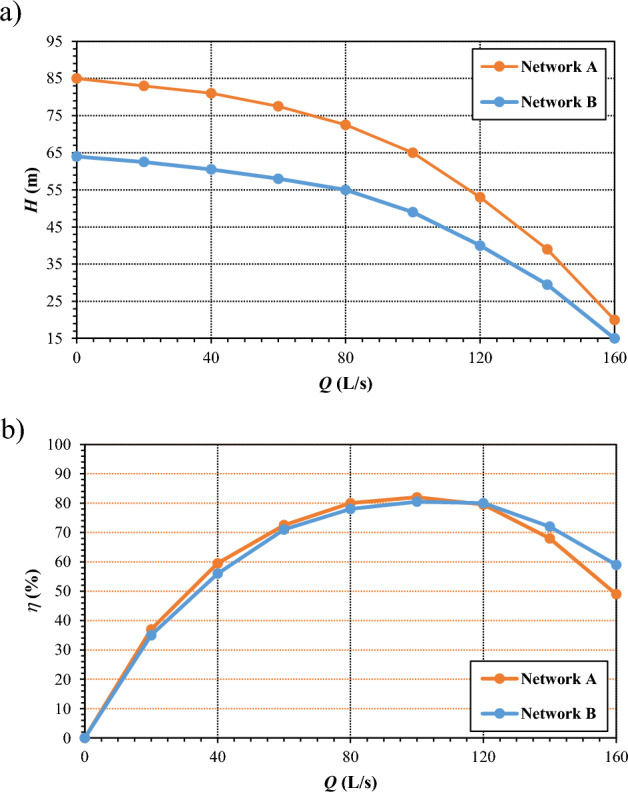
Characteristic curves for the pumps in Networks A and B: (**a**) discharge *Q* – head *H* and (**b**) discharge *Q* – efficiency *η*.

The pumping station was modelled assuming the three ordinary pumps to be active at the maximum frequency of 50 Hz in both Network A and Network B. Based on the information provided by the Consortium, the pump model considered for the three ordinary pumps in Network A is the Caprari P14CS/8/40/4D. The model considered for the three ordinary pumps in Network B is the Caprari P14CF/8/40/3D. The characteristic curves of both models are reported in Fig. 6.

### Optimization of the network

The optimization of the pipe diameters was carried out in the phases 1 and 2 of work aimed at redesigning the network, by using a bi-objective genetic algorithm implemented in the Matlab® 2023a environment. The genetic algorithm is an adaptive mathematical procedure that solves optimization problems by mimicking living species’ evolution and adaptation to the surrounding environment by means of the cross-over and mutation processes^23^. The solutions inside the genetic algorithm are encoded in a population of *pop* individuals, each of which is made up of as many genes as the decision variables in the optimization problem.

At the beginning of the application of the genetic algorithm an initial population is generated, to be evolved over generations. In the generic *k*-th generation, children are generated from parents by means of the crossover and mutation processes to create the population for the following *k* + 1-th generation. The steps of the genetic algorithm are iterated over generations till no significant improvements are remarked in the population’s fitness. The ultimate solution of the genetic algorithm is made up of a Pareto front of optimal trade-off solutions between the objective functions considered in the optimization.

By following the default settings in Matlab® 2023a, a population *pop* of 200 individuals was used in this work. Furthermore, the crossover and mutation percentages were set to 80% and 20%, respectively.

In the present case study, the first objective function to minimize was the total cost of network pipes, obtained by means of the following formula:

1 $$C=\sum_{i=1}^{p}{c}_{i}{L}_{i}$$

in which *c* (€/m) and *L* (m) are the unit costs and pipe lengths, respectively, while *p* is the total number of pipes, equal to 483 and 513 in the phases 1 and 2 of work, respectively. In fact, as was described in section “Introduction”, phase 2 considered the presence of 30 additional pipes compared to phase 1, to close the external loops of the network. In the cost *C*, the expenses for disposing of the current network in asbestos cement were neglected, as well as the costs for the installation of isolation/control devices in the new network and for land expropriation to enable pipe laying on new sites not considered in the current layout.

The unit cost of the new pipes was considered to be a growing function of the diameter, as is shown in the following Table 1, derived from the regional price list for PEAD PN16 elements.

**Table 1:** Unit costs *c* for the pipe as a function of the external diameter *D*_*e*_ and of the internal diameter *D*_*i*_ for PEAD PN16 pipes.

*D*_*e*_ (mm)	*D*_*i*_ (mm)	*c* (€/m)
125	102.2	13.42
200	163.6	24.55
250	204.6	34.95
355	290.6	66.61
500	409.2	123.06
630	515.6	194.9

As the second objective function to maximize in the optimization process, the minimum pressure head at nodes with outflow was calculated using the following formula:

2 $${h}_{min}=\mathrm{min}\left({h}_{si,j}\right)$$

in which *h*_*si,j*_ (m) is the pressure head at the *i*-th node with outflow in the *j*-th peak demand scenario.

The pair of objective functions composing the fitness was evaluated for each of the individuals generated by the genetic algorithm. Notably, the calculation of the second objective function in Eq. (2) was carried out by hydraulically solving the network with pipe diameters modified based on individual genes in the genetic algorithm, by means of the EPANET 2.2 toolkit^24^.

Inside the bi-objective genetic algorithm, a number of decision variables equal to *p* was considered. Each decision variable took on an integer value between 1 and 6, to indicate which of the six pipe diameters reported in Table 1 was assigned to the corresponding network pipe.

In the optimization, *n*_*c*_ linear constraints expressed as follows were considered:

3 $$\mathbf{A} \mathbf{D}\le 0$$

in which **D** (*p* × 1) is the vector of pipe diameters. Matrix **A** (*n*_*c*_ × *p*) is a matrix with elements equal to 0, − 1 or 1, in which the *n*_*c*_ rows are associated with the nodes with outflow in the network. For the *k*-th constraint associated with one of the nodes with outflow, all elements are set to 0, except for *A*(*k*,*l*) = 1 and *A*(*k*,*m*) = − 1, with *l* and *m* representing the indices associated with a single pipe downstream and a single pipe upstream from the node, respectively. The matrix expression (3) entails that *D*_*l*_ ≤ *D*_*m*_, i.e., that the generic pipe downstream of a node with outflow cannot have a larger diameter than the generic pipe upstream from the node. The presence of the constraints in Eq. (3) enforces the telescopic property in each individual in the optimization. As a result, for all individuals in the genetic algorithm, the diameters get progressively smaller while traversing the network from the source towards external nodes, consistently with engineering judgment. In the present work, the distinction between upstream and downstream pipes for each node with outflow was obtained based on the pipe water discharge values in the network configuration obtained by assigning the largest diameter in Table 1 to all network pipes.

## Results

**Figure 7:**
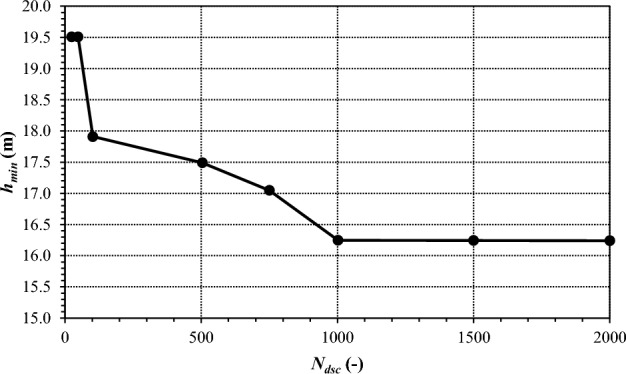
*h*_*min*_ as a function of *N*_*dsc*_ for the current network.

As a preliminary analysis before Phases 0, 1 and 2, the choice of the number *N*_*dsc*_ of demand scenarios to consider for the analysis of the performance of the current and redesigned network was made by testing how results in terms of meaningful hydraulic indicators are affected by changing *N*_*dsc*_. Taking the current network as benchmark and considering *h*_*min*_ defined in Eq. (2) as the meaningful hydraulic indicator, the iterated application of the software EPANET 2.2 yielded the results shown in the following Fig. 7 in terms of *h*_*min*_ as a function of *N*_*dsc*_. Starting from values around 19.5 m, *h*_*min*_ tends to decrease as *N*_*dsc*_ grows. However, for *N*_*dsc*_ ≥ 1000, the values of *h*_*min*_ appear to be stabilising around 16.2 m, proving that the hydraulic performance of the network can be satisfactorily assessed considering a sample of *N*_*dsc*_ = 1000 scenarios. In fact, larger samples do not give additional contribution to the assessment of the hydraulic performance, while increasing the computational burden. Therefore, the representative sample of *N*_*dsc*_ = 1000 scenarios was considered in the Phases 0, 1 and 2 of the work, described below.

### Phase 0

**Figure 8:**
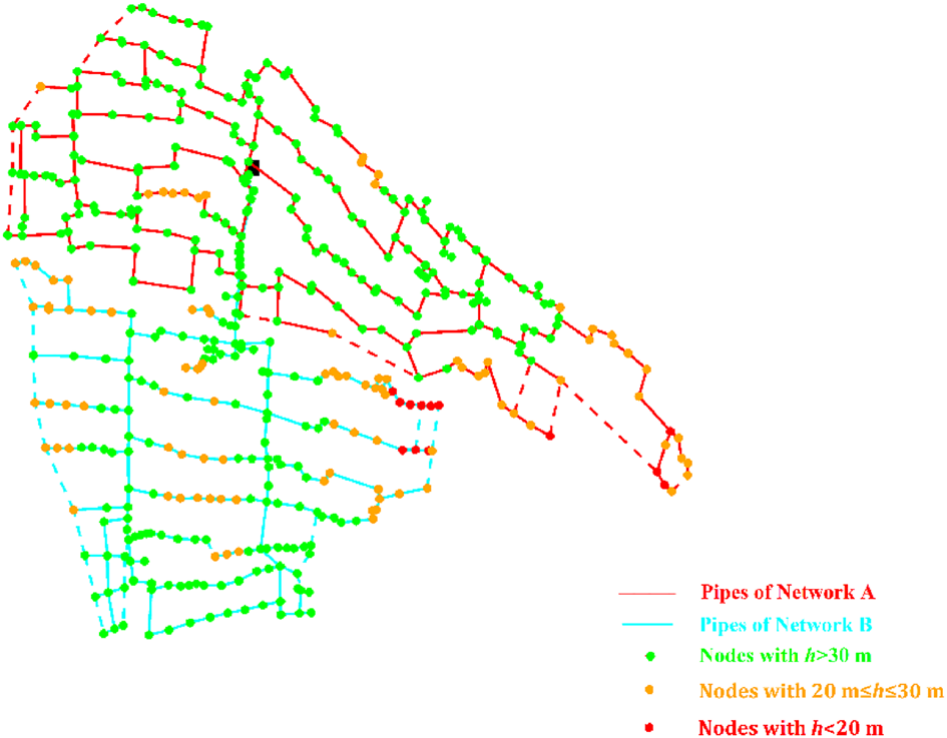
Current network with open external loops (dotted lines associated with not installed pipes) and minimum nodal pressure heads in peak demand scenarios.

The application of the software EPANET 2.2 to the current network (phase 0 of work) yielded the results reported in terms of minimum nodal pressure heads in the peak demand scenarios in the following Fig. 8. This figure differentiates between nodes with fully satisfactory pressure heads (> 30 m), nodes with barely sufficient pressure heads (≥ 20 m and ≤ 30 m) and nodes with insufficient pressure heads (< 20 m). The results confirm the presence of numerous pressure deficits below 30 m, as was remarked during *in-situ* observations, therefore justifying the need for network redesign.

### Phases 1 and 2

**Figure 9:**
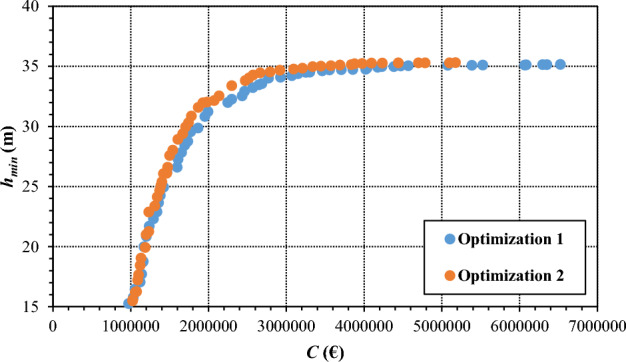
Pareto front of optimal trade-off solutions between total cost *C* and minimum pressure head *h*_*min*_ in the network, obtained in optimization 1 (phase 1) and optimization 2 (phase 2), considering the network configurations with open and closed external loops, respectively.

The results obtained for network redesign after about 100 generations of the bi-objective genetic algorithm in phases 1 and 2 of work are shown in the graph in Fig. 9, in terms of Pareto fronts between cost *C* and minimum pressure head *h*_*min*_ in the network. As expected, this graph shows, for both the Pareto fronts, growing values of *h*_*min*_ as the cost *C* increases. This happens because it takes the enlargement of pipe diameters to reduce head losses and to increase service pressure in the network, the head supplied by the pumping station being equal. The total cost *C* being the same, the Pareto front of optimization 2, related to phase 2, offers better solutions in terms of *h*_*min*_ than the Pareto front of optimization 1, related to phase 1. Though incurring additional expenses for pipe installation on new sites, the closure of the external loops in phase 2 enables money savings in the diameters used in other parts of the network, therefore resulting in better hydraulic performance with equal total cost. This confirms the results obtained in^7–9^ about the benefits of topological redundancy.

**Figure 10:**
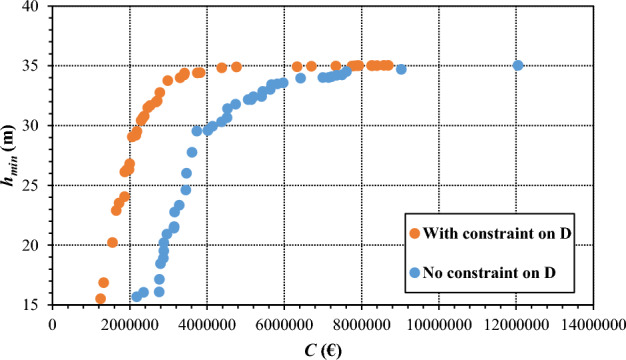
Optimization 2. Pareto fronts of optimal trade-off solutions between total cost *C* and minimum pressure head *h*_*min*_ in the network, obtained after 10 generations of the genetic algorithm in the presence and absence of the constraints on pipe diameters.

To give evidence about the numerical benefits of the methodology adopted, the results reported in Fig. 10 point out that, after 10 generations of the genetic algorithm in optimization 2, the adoption of the linear constraints in matrix Eq. (3) on pipe diameters for the enforcement of the telescopic property in the network speeded up the convergence towards effective solutions from the engineering viewpoint, in comparison with the solutions obtained by applying the algorithm without these constraints. In fact, the graph in Fig. 10 shows that the adoption of the constraints results in solutions with a much lower cost *C*, the value of *h*_*min*_ being equal.

The choice of the ultimate solutions in optimizations 1 and 2 was carried out to limit the budget and by considering the position of the knee in the Pareto fronts shown in Fig. 9, around 2,000,000 €. This budget results in solutions capable of guaranteeing fully satisfactory service pressure heads, that is significantly larger than 30 m at all nodes and in all peak demand scenarios.

The following Table 2 reports the costs of the ultimate solutions 1 and 2, obtained in phases 1 and 2 of work with a budget very close to 2,000,000 €. The analysis of the table points out that Network A demands the larger investment in both the solutions, because of the larger overall length and more unfavorable altitude conditions.

**Table 2:** Costs in millions of € for the two design solutions, for the high-altitude network (Network A), the low-altitude network (Network B) and for the overall network.

Design solution	Network A	Network B	Overall
1	1.2043	0.82436	2.0286
2	1.2014	0.83457	2.0360

The following Table 3 reports the network lengths associated with the external diameters used for network design (see Table 1). Design solution 2 makes wider use of the smallest diameters 125 mm and 200 mm and prevents installation of the largest diameter 630 mm. This results in a total cost almost equal to that of design solution 1, despite the larger length extension.

**Table 3:** Length *L* of network associated with the various external diameters *D*_*e*_.

*D*_*e*_ (mm)	*L* (m) for Design solution 1	*L* (m) for Design solution 2
125	10,687	17,752
200	13,696	16,398
250	18,352	18,585
355	9258	6624
500	2106	2474
630	164	0
All diameters	54,263	61,834

**Figure 11:**
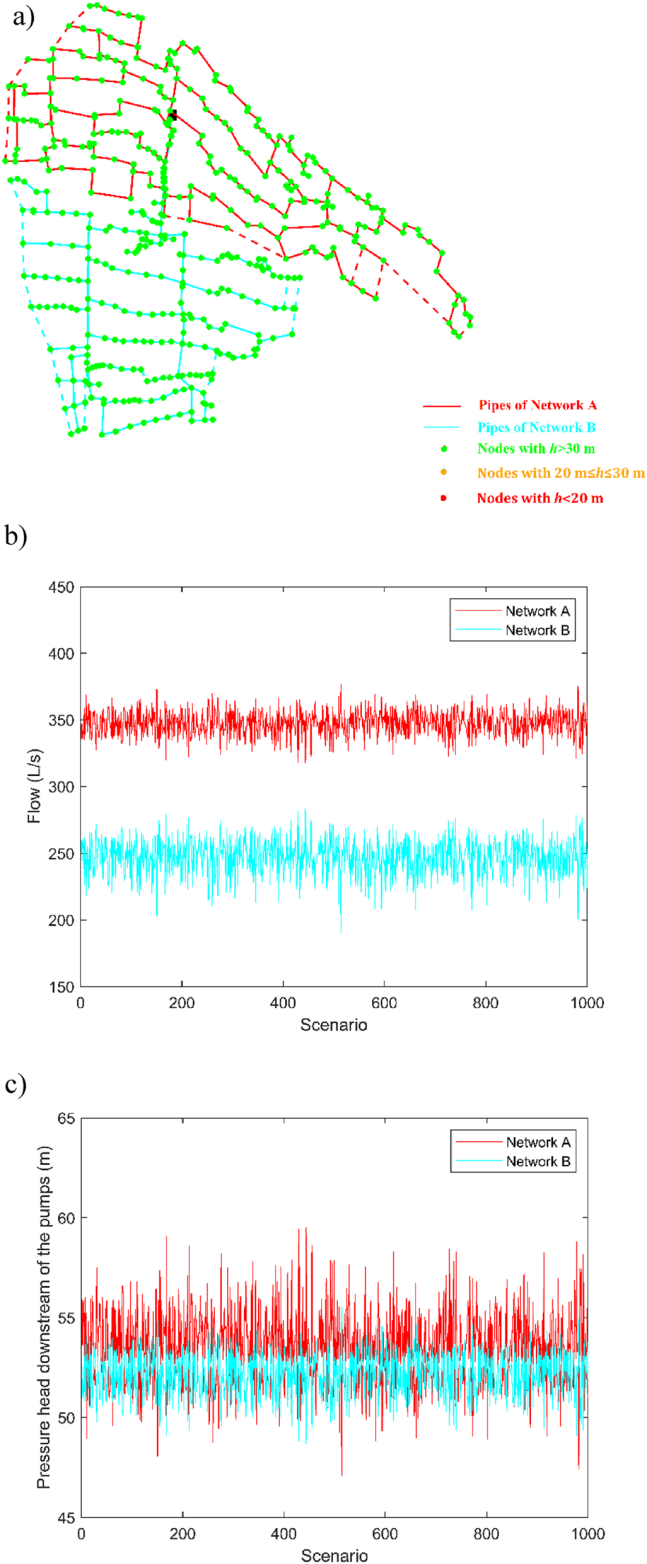
(**a**) Design solution 1 with open external loops (dashed lines corresponding to not installed pipes) and minimum nodal pressure heads in the peak demand scenarios; (**b**) pumped water discharges and (**c**) service pressure heads downstream of the pumping station for Network A and Network B in the peak demand scenarios.

**Figure 12:**
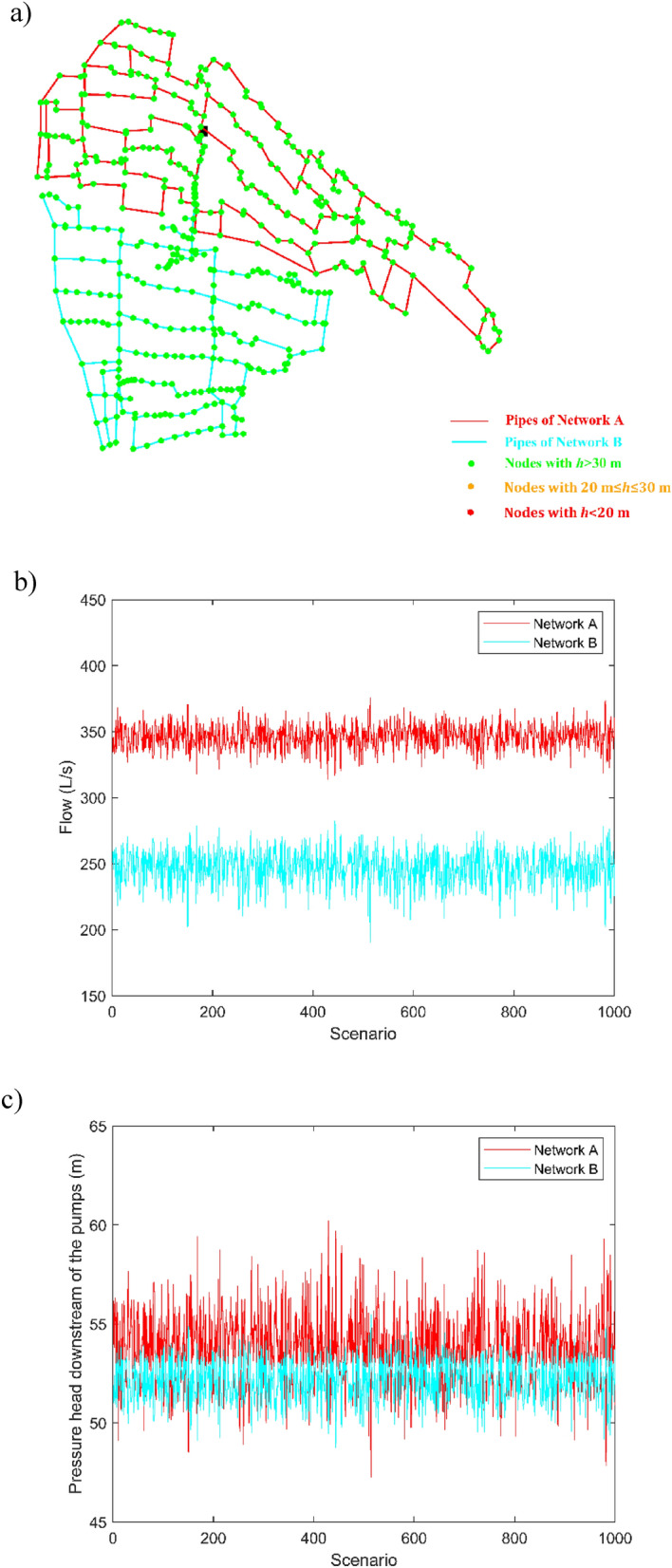
(**a**) Design solution 2 with closed external loops and minimum nodal pressure heads in the peak demand scenarios; (**b**) pumped water discharges and (**c**) service pressure heads downstream of the pumping station for Network A and Network B in the peak demand scenarios.

The following Figs. 11 and 12 report some meaningful results for design solutions 1 and 2, respectively, in terms of nodal pressure heads (Figs. 11a and 12a), pumped water discharges (Figs. 11b and 12b) and pressure heads downstream of the pumping station (Figs. 11c and 12c) in the 1000 peak demand scenarios.

Notably, Figs. 11a and 12a prove that both the solutions can guarantee fully satisfactory nodal pressure heads. Figures 11b and 12b point out that the pumped water discharge in Networks A and B lies around 350 L/s and 250 L/s, respectively. The sum of the values is around 600 L/s, therefore abiding by the concession water discharge under peak conditions and rendering superfluous the carrying out of flow balance processes in the tank upstream from the Telgate station. Finally, Figs. 11c and 12c point out that the service pressure downstream of the pumping station in the peak demand scenarios lies around 54 m and 53 m, for Network A and Network B, respectively. These values can also be kept as target settings in less demanding scenarios, when the number and rotational speed of active pumps are lower.

## Conclusions

The present work concerned the design of the pressurized irrigation network serving an area of about 750 ha in Northern Italy and was carried out by using a methodology consisting of:

- Construction of peak demand scenarios, by randomly selecting the installation sites for available hydrant heads at each sector of irrigated properties.
- Pressure-driven hydraulic modelling at hydrant sites.
- Bi-objective genetic optimization of pipe diameters for network design in the current network layout (optimization 1) and in another layout (optimization 2) modified by considering additional pipes for the closure of external loops.

The first two methodological elements enabled the snapshot performance of the existing and optimized networks to be modelled realistically in networks serving properties grouped into sectors. Thanks to the implementation of constraints for the enforcement of the telescopic property in the network, i.e., the diameter reduction from internal to external areas, the third methodological element yielded effective design solutions in both the network layouts considered. The comparison of the ultimate design solutions pointed out a better hydraulic performance for the solution in optimization 2, which is also more reliable. In fact, it offers more numerous potential water paths to reach demanding nodes during mechanical failure events (pipe bursts). Nevertheless, the adoption of solution 2 entails some legal problems, due to the need for land expropriation to install pipes on new sites, which need to be considered by the Consortium in the choice of the ultimate solution.

## Data Availability

The datasets used and/or analyzed during the current study available from the corresponding author on reasonable request.
